# Bioinspired antifouling Fe-based amorphous coating via killing-resisting dual surface modifications

**DOI:** 10.1038/s41598-021-04746-y

**Published:** 2022-01-17

**Authors:** Yu Li, Ling-Yu Zhang, Cheng Zhang, Zhan-Rong Zhang, Lin Liu

**Affiliations:** State Key Laboratory of Materials Processing and Die and Mold Technology, School of Materials Science and Engineering, Huazhong University of Science and Technology, Wuhan, 430074 China

**Keywords:** Biomaterials, Nanoscale materials, Structural materials

## Abstract

Fe-based amorphous coatings with outstanding corrosion resistance are promise for marine applications. However, these coatings encounter a great challenge of biofouling in marine environments. Inspired by the unique micro-nano hierarchical structure of shark skin with excellent antifouling properties, in this paper, we construct a bioinspired Fe-based amorphous coating with killing-resisting dual-effect via proper surface modifications, i.e., the modification with micro-patterned nanostructured Cu_2_O fibers (killing effect), followed by the modification with superhydrophobic surface (resisting effect). As a result, the modified amorphous coating exhibits impressive antifouling properties, achieving 98.6% resistance to *Nitzschia closterium f. minutissima,* 87% resistance to *Bovine serum albumin* protein and 99.8% resistance to *Pseudomonas aeruginosa*, respectively. The remarkable antifouling performance is attributed to a synergistic antifouling mechanism from both resisting effect and killing effect, wherein the superhydrophobic surface provides a barrier to resist protein adsorption, while the patterned nanostructured Cu_2_O fibers supply Cu^+^ ions to kill bacterial cells. In addition, the modified amorphous coating also exhibits excellent mechanical robustness, which ensures the durability of the Fe-based amorphous coating in practical services. This work may promote the development of new durable metal-based coatings integrated with anti-fouling and anti-corrosion properties.

## Introduction

Marine fouling, termed as the colonization on submerged surfaces by marine micro-organisms, has been a long-standing global problem^1–7^. In spite of substantial efforts on this subject for decades, creating robust antifouling surfaces is still challenging. Currently, two antifouling approaches are proposed: The first one relies on the release of biocides to kill marine organisms; the other one is based on coating itself for resisting organism adhesion, such as self-polishing and self-fouling-releasing^3,5^, through specific surface modifications^2,7–9^. However, for the first approach, the uncontrollable release rate of biocides and the accumulation of the dead bacteria or proteins significantly reduces the antifouling efficiency^10^; for the second approach, the poor mechanical robustness of the modified surfaces easily leads to the loss of the anti-fouling functions due to wear or impact^11,12^. In addition, the weak adhesion of polymer coatings to metallic substrates (typically < 3 MPa)^13^ limits the durability of the coatings. Accordingly, numerous efforts have been devoted to explore alternatives to replace the polymer-based antifouling coatings. From the viewpoint of usability and durability, one effective approach is to develop metal-based antifouling coatings. Huang et al.^14^ fabricated a two-layered Cu/Al_2_O_3_ composite coating via cold spray, which exhibits a high adhesion strength (~ 20 MPa) to a steel substrate and good anti-biofouling resistance (85%) against barnacles, diatoms and mussels. Recently, Tian et al.^15^ developed a plasma-sprayed Cu-Ti coatings with microscale Cu/Ti laminated microstructure on a steel substrate, which also demonstrates excellent antifouling properties against *Escherichia coli (E. coli)* bacteria because of Cu ions release and self-polishing effect. Unfortunately, the corrosion resistance of these coatings is not satisfactory, limiting the durability in services.

Regarding the marine applications, the coating should have two functions: anti-corrosion and anti-fouling. In recent years, Fe-based amorphous coatings have attracted widespread interest because of their excellent corrosion resistance in aggressive environments, which is superior to other conventional crystalline coatings such as thermally-sprayed stainless steel coatings and electroplated Cr coatings, demonstrating a promise potential in marine applications^16–27^. In addition, the amorphous coatings typically have high hardness (above 1000 Hv), good wear-resistance, and high adhesion strength (up to 40 MPa) to various metallic substrates^17^ that far exceeds the polymer-based coatings. However, in practical marine applications, except for the requirement of corrosion, bio-fouling is another important concern^28^. So far, little work has been done on this subject regarding Fe-based amorphous coatings. Given that the Fe-based amorphous coatings readily possess high corrosion resistance, modifying them with antifouling feature will provide these metal-based coatings with integration of both anti-fouling and anti-corrosion properties.

**Figure 1:**
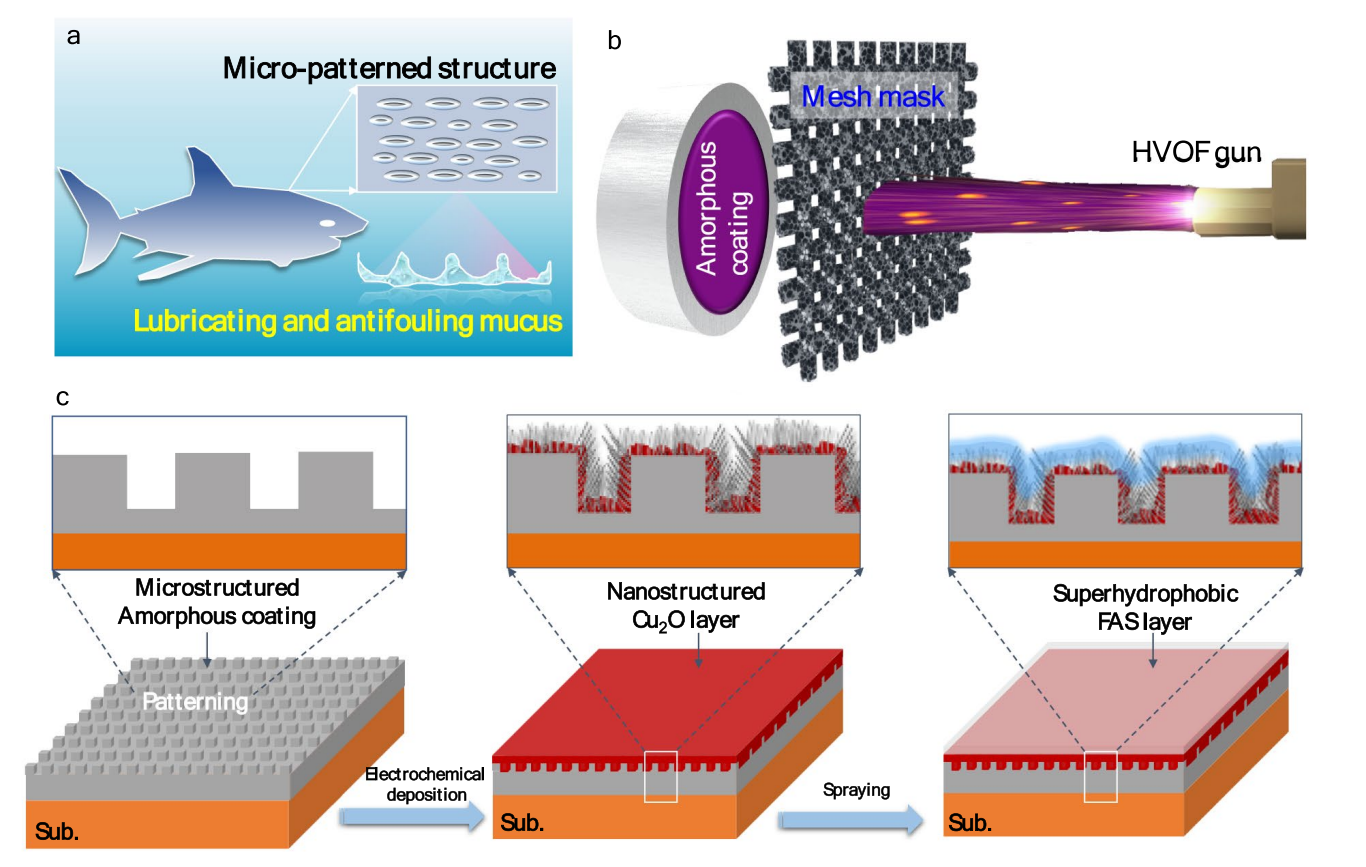
(**a**) A carton picture showing the micropatterned surface of shark skin, which also continuously releases lubricating and antifouling mucus. (**b**, **c**) Schematic illustration of the preparation of shark skin -inspired Fe-based amorphous coating via dual surface modifications. Firstly, a micro-patterned amorphous coating with a thickness of about 100 μm was prepared using mask-assistant thermal spray technique. The mask, made of Fe-wire mesh with a single square pore size of around 400 × 400 μm, was put between the spraying gun and the substrate. Secondly, a layer of nanostructure Cu_2_O fibers was deposited on the as-sprayed coating via electrochemical deposition followed by cycling CV treatment. Finally, the sample was coated with a FAS layer to achieve superhydrophobicity.

Fouling by marine micro-organisms is affected by various aspects of materials, including chemical composition, wettability, surface charge, roughness and surface mechanical properties (i.e., elastic modulus)^29^. Modification towards these aspects thus allows us to modulate the antifouling properties of the materials. Although marine fouling is complex, nature has created multiple strategies to minimize nonspecific adhesion^30^. One representative example is from shark skin, which has intrinsically a unique micro-nano hierarchical structure, and enables to continuously release lubricating and antifouling mucus, so shark shin demonstrates excellent antifouling properties^30^. Inspired by the natural characters of shark skin (Fig. 1a), in this work, we designed and fabricated a novel bioinspired antifouling Fe-based amorphous coating surface via dual surface-modifications, i.e., the modification with micro-patterned nanostructured Cu_2_O fibers, followed by the modification with a superhydrophobic layer, which endows the Fe-based amorphous coating with both killing and resisting functionality, thus significantly enhance the antifouling properties.

## Results and discussion

To realize a robust superhydrophobic surface, the key issue is to construct a micro/nano hierarchical structure surface, where the nanostructure provides water repellency and the microstructure provides mechanical durability^31^. Figures 1b,c schematically illustrates the preparation of Fe-based amorphous coatings with micro/nano hierarchical structures. On the micrometric length scale, a micro-patterned Fe-based amorphous coating was fabricated via a mask-assistant high-velocity oxygen fuel (HVOF) thermal spray technique. On the nanometric length scale, a nanostructured Cu_2_O layer was deposited on the as-sprayed amorphous coating via electrochemical deposition/cyclic voltammetry (CV) treatment coupled techniques^32^. Cu_2_O is chosen here because it is widely used in the antifouling industries^33^. Finally, a low-surface-energy layer of FAS (C_13_H_13_F_17_O_3_Si) was coated on the above modified coating, resulting in a superhydrophobic surface that could resist the adhesion of organisms. Notably, this design strategy has multifold functions, the nanostructured Cu_2_O layer serves as fouling-release agent via gradually release of Cu^+^ in seawater^34^ on one hand, while the FAS layer controls the release rate of Cu^+^ ions and increases the durability of the coating on the other hand.

**Figure 2:**
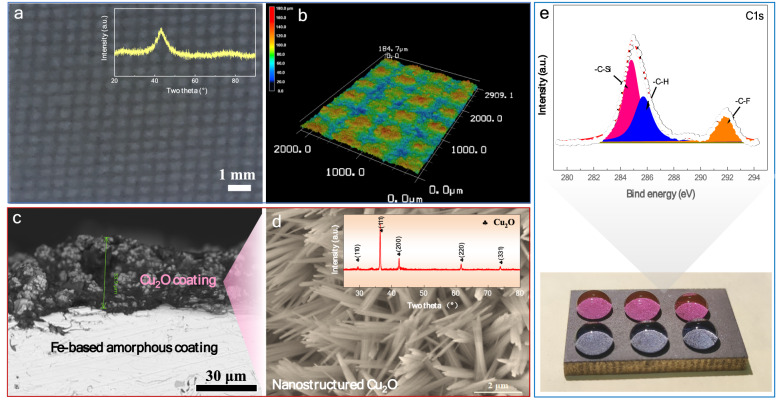
Structure of the as-prepared coating. (**a**) Surface morphology and (**b**) 3D surface topography on the as-sprayed and micro-patterned Fe-based amorphous coating. Inset in (**a**) shows the XRD pattern of the coating before modification. (**c**) Cross sectional and (**d**) surface morphologies of the coating after deposition of Cu_2_O layer. Inset in (**d**) is the XRD pattern of the Cu_2_O layer deposited on amorphous coating. (**e**) Photo of FAS-Cu_2_O modified amorphous coating and XPS spectra from the surface.

The surface morphology of as-sprayed Fe-based amorphous coating (termed as coating P) is presented in Fig. 2a and b, which shows a well-defined pillar-like pattern with diameter of approximately 450 μm and height of about 165 μm. The X-ray diffraction (XRD) pattern (see the inset in Fig. 2a) indicates that the coating is of a single-phase amorphous structure. Upon electrochemical deposition, a porous Cu_2_O layer with a thickness of around 34 μm was successfully deposited on the amorphous coating, as evidenced from the cross-section morphology in Fig. 2c. Notably, the as-deposited Cu_2_O layer exhibits particle-like morphology, which was reformed to nanofibers (see Fig. 2d) through CV electro-oxidation treatment. The individual Cu_2_O nanofiber has a dimension of 2 μm in length and less than 20 nm in diameter. The purpose of making Cu_2_O nanofiber is to obtain a nano-structure, which is the requisition of superhydrophobicity^31^. XRD verifies that the Cu_2_O nanofibers are of FCC structure (Fig. 2d). Therefore, a micro/nano hierarchical structure was successfully constructed on the Fe-based amorphous coating. Finally, to realize superhydrophobicity, the Cu_2_O-modified sample was further coated with a FAS layer (low surface energy) to achieve high water repellency (Fig. 2e). X-ray photoelectron spectroscopy (XPS) examination indicates the appearance of C-Si and C-F bonding on the coating surface (Fig. 2e), confirming that FAS molecule present at the surface. According to previous works^35,36^, the first FAS molecule layer could bond strongly with oxygen atoms and/or surface hydroxyl groups on the substrate via chemical bonding, which cannot be easily removed by mechanical rubbing or washing by acetone or propanol. Hereafter, the amorphous coatings modified with Cu_2_O nanofibers, FAS layer, and dual-layers of Cu_2_O nanofibers and FAS, are termed as coating PC, coating PF and coating PCF, respectively.

**Figure 3:**
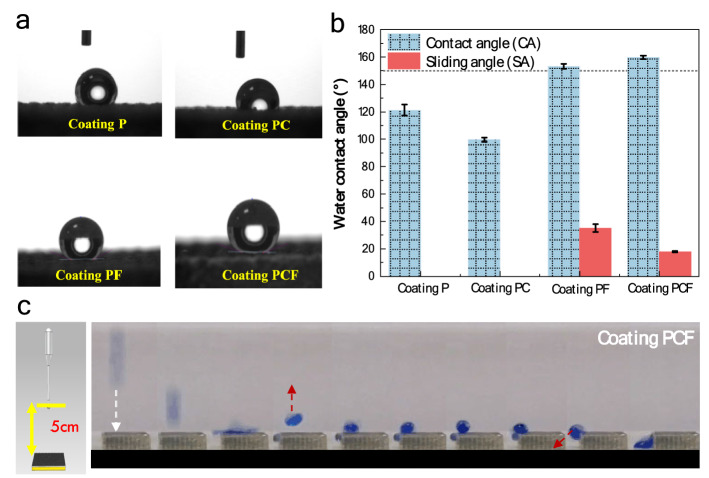
Surface wettability of as-modified amorphous coatings. (**a**) The contact of water droplet on the 4 different coatings, including as-patterning coating (coating P), the coating modified only with Cu_2_O (coating PC), the coating modified only with FAS (coating PF), and the coating with dual modifications (coating PCF). (**b**) Water contact angle (CA) and sliding angle (SA) of the coatings. (**c**) The images illustrate water droplets bouncing on the coating PCF.

The wettability of the coating PCF was examined by water contact angle (WCA) and sliding angle (SA) measurements using water as the testing liquid, and the results are compared with coating P, coating PC and coating PF. As shown in Fig. 3a and b, the coating PF and coating PCF exhibit a WCA above 150°, indicative of a superhydrophobic feature. However, the coating PCF shows a much lower SA (~ 20°) than the coating PF (~ 35°), demonstrating that the former has a better water repellency property. By contrast, the coating PF and coating P without FAS layer have a WCA between 100 and 120° and water droplet could not be able to slip, indicating non-superhydrophobicity. Furthermore, the droplet impact tests on the coating PCF surface clearly shows a good rebounding behavior (Fig. 3c), demonstrating that this coating exhibits good superhydrophobicity. The water bouncing performance is a typical hallmark of good water-repellent surfaces^37^.

**Figure 4:**
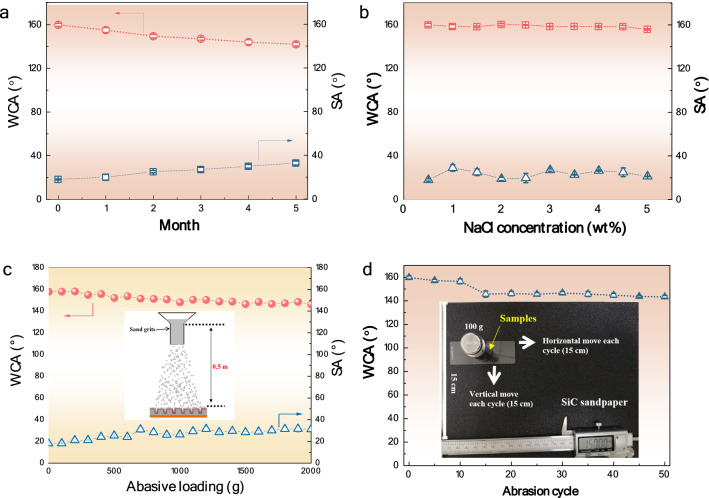
Chemical stability and mechanical robustness of the coating PCF. (**a**) Water contact angle (WCA) and sliding angles (SA) measured on the coating after different months. (**b**) WCAs and SAs measured with NaCl solution droplet with different concentrations. (**c**) WCAs and SAs measured after the coating PCF suffered from different amounts of Al_2_O_3_ sand abrasive loading (inset schematically illustrates the sand abrasion test). (**d**) WCAs variations of the coating PCF with sandpaper abrasion cycles (inset shows the technical model of the sandpaper abrasion test).

In practice applications, the coatings may encounter different complex and harsh environments, which may lead to the loss of superhydrophobicity of the coating due to the chemical and/or mechanical damage^31^. To this end, we evaluated the robustness of the coating PCF in different conditions, such as corrosion, sandpaper abrasion and sand particle erosion tests. Figure 4a shows the variations of CAs and SAs in open air for different times. It is seen that the coating remains superhydrophobicity with WCA > 150° and SA < 30° within 3 months. To assess chemical stability, the wettability of the coating PCF was examined using NaCl solution droplets with different concentrations. As shown in Fig. 4b, the CA remains greater than 150°, while the SA is smaller than 30° even in high saline condition (i.e., 5 wt.%), demonstrating that the coating has a good chemical stability in corrosive environments. The mechanical robustness of the coating PCF was tested by continuous impacting with Al_2_O_3_ sand particles (0.7–0.85 mm in size) from a height of 0.5 m (inset in Fig. 4c), corresponding to an impacting speed of 3.13 m/s. Figure 4c shows that the CAs and SAs of the coating PCF remain nearly unchanged (CA > 150°, SA < 35°) upon different abrasions, indicating that this coating is sufficiently robust. In addition, the sandpaper abrasion tests were also performed (see Fig. 4d). It shows that the coating PCF keeps strong hydrophobicity with a high CA of approximately 144° after 50 abrasion cycles under an applied pressure of 9800 N m^−2^. The outstanding mechanical robustness results from the robust micro/nano hierarchical structure, which keeps the coating with good hydrophobicity even though the upper layer of Cu_2_O nanostructures are partially peeled off.

**Figure 5:**
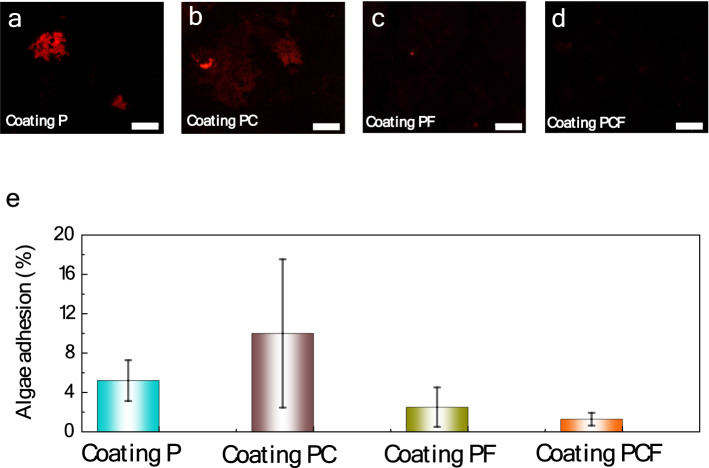
Anti-algal adhesion performance. Fluorescent images of *Nitzschia closterium f. minutissima* adsorption on (**a**) coating P, (**b**) coating PC, (**c**) coating PF and (**d**) coating PCF, after immersion for 15 days. (**e**) The algal adhesion on the 4 different coatings.

The antifouling properties of the coatings were evaluated via algal adhesion tests, using the *nitzschia closterium f. minutissima* as the model algae. Figures 5a–d show the fluorescence micrographs of the adherent algae on the four different coatings after immersion in the solution for 15 days. It is evident that the algae are visibly adhered on coating P and coating PC (i.e., without surface modification or only with Cu_2_O modification), indicating a poor resistance to algae adhesion and biofilm growth. Contrarily, very few algae were observed on coating PF and coating PCF (both are with superhydrophobic modification), demonstrating an excellent antifouling ability of the latter two coatings. Based on statistical analysis over five different fluorescence images, the averaged algae adhesion in terms of ratio of the area covered with the biofilm was calculated to be 5.2%, 10.0%, 2.5% and 1.2% for the coatings P, PC, PF and PCF, respectively (Fig. 5e). This result reveals that the two superhydrophobic coatings (i.e., coating PF and coating PCF) show higher resistance against algae growth than the one without modification or with only Cu_2_O modification, and the coating PCF with dual surface-modifications demonstrates the best antifouling ability. The relatively poor anti-algae adhesion of coating PC is related to its lowest water contact angle, and the structural change of the outmost Cu_2_O layer during long-term immersion due to the so-called microbiologically influenced corrosion (MIC) effect. Indeed, after 15 days of immersion, partial of the surface on coating PC turns to be aeruginous, indicating the formation of copper hydroxide. Reasonably, the Cu(OH)_2_ has a reduced antifouling property than Cu_2_O. The presence of a superhydrophobic FAS layer, furthermore, not only reduces the adhesion of algae, but also controls the Cu^+^ ion release rate, thus reducing MIC effect and improving fouling resistance in long-term immersion. To verify experimentally the influence of FAS layer on Cu^+^ ion release, we carried out the Cu^+^ ion measurement on the Fe-based amorphous coatings with and without FAS modification after immersion in deionized water for different times. The results show that, for coating PC (without FAS), the Cu^+^ release rate is 28.7 and 55.7 μg cm^−2^ day^−1^, respectively, after 4 and 7 days of immersion, respectively. By contrast, for the coating PCF (with FAS), the Cu^+^ ion release rate was sharply reduced to 1.30 and 1.90 μg cm^−2^ day^−1^ after the same period of immersion. It is suggested that the controllable release of Cu^+^ ion plays an important role in improvement of durability of the dual-modified amorphous coatings.

**Figure 6:**
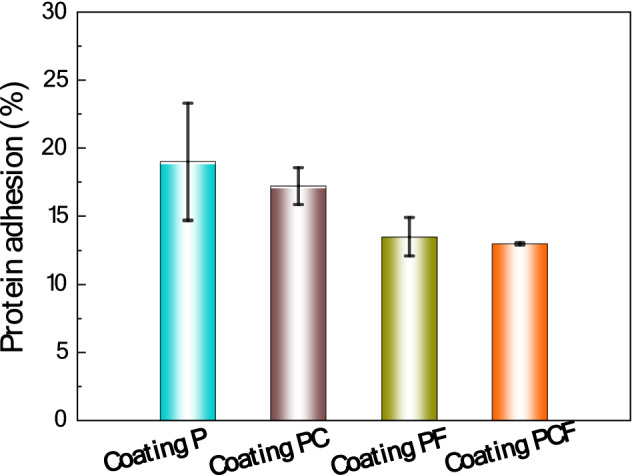
The BSA protein adhesion on the 4 different coatings.

In general, marine biofouling starts with release of proteins from bacteria onto the target surface, the accumulation of proteins provides a platform for bacterial colonization and biofilm formation. The slime forming algae (diatoms) then colonize the biofilms and finally results in microfouling^38,39^. Therefore, the ability to retard the adsorption of proteins and bacterial adhesion on coating surface is a measure to evaluate the quality of antifouling coatings. To this end, the anti-protein-fouling and anti-bacterial adhesion performance of the four coatings studied in this work were assessed. Here, the anti-protein-fouling properties were examined using the *Bovine serum albumin* (BSA) as the model protein. The result in Fig. 6 reveals that, after immersion in the BSA for 1 day, the coatings PCF and PF show higher resistance (87%) to protein adhesion than the coating P (81%) and coating PC (83%), which is contributed to the resisting effect of the modified amorphous coating that prevents adhesion and accumulation of proteins on the coating surface, because the superhydrophobic FAS-modified surface reduces the opportunities for H-binding and polar interactions with adhesive proteins.

**Figure 7:**
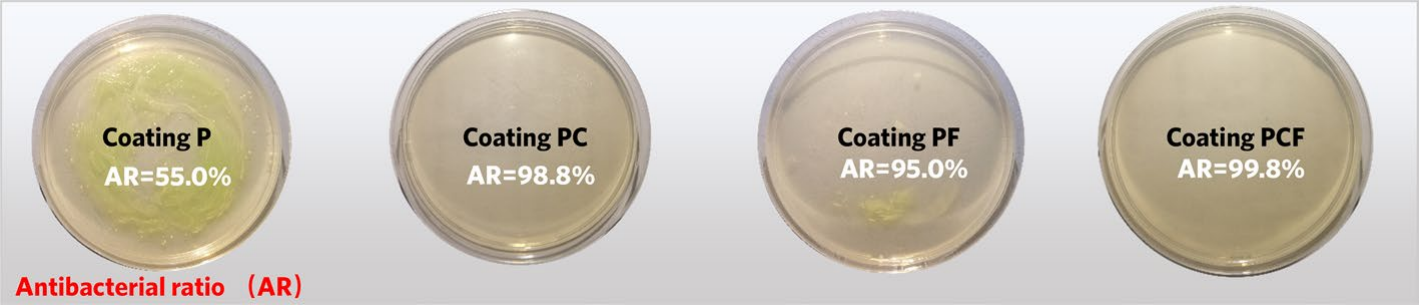
Antibacterial property of the coatings against a marine bacteria *Pseudomonas aeruginosa.*

In addition, we further evaluated the antibacterial efficiency of the four different coatings since bacterial adhesion is a critical step during biofilm formation^40^. Figure 7 shows the antibacterial property of different coatings against a representative marine bacteria *Pseudomonas aeruginosa.* It is seen that coating PC and coating PF (modified with Cu_2_O and FAS) has much less bacteria colonies than coating P (without any modifications). Specially, the coating PC exhibits better antibacterial property than coating PF due to the killing effect of Cu^+^ ion in the former^41^. The Gram-negative *Pseudomonas aeruginosa* possesses only a 2.4 nm cell wall^42^, such that Cu^+^ could move into its cell and leads to cytoplasmic outflow and cell membrane destruction. Indeed, Li et al.^33^ has observed a large amount of metallic copper element inside the cell of *E. coli* with 7–8 nm cell wall. Furthermore, the coating with dual-modifications displays the best antibacterial performance, which shows the highest antibacterial ratio (99.8%). The combination of the killing effect (owing to Cu^+^ release) and resisting effect (owing to surface superhydrophobicity) makes it difficult for bacteria to form bacterial film on the surface of the coatings, which is therefore not conductive to micro-organisms adhesion.

**Figure 8:**
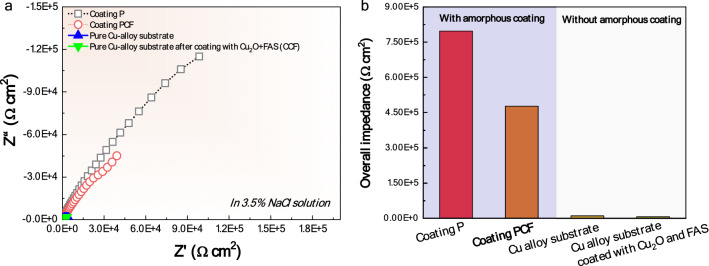
Corrosion resistance of the coatings in a 3.5% NaCl solution. (**a**) Nyquist plots of the coating PCF, compared with the unmodified coating P, pure CuAl alloy substrate. (**b**) Comparison of the overall impedance of the four samples, highlighting the importance of the Fe-based amorphous coating in enhancing the corrosion resistance.

Finally, as marine biofouling and corrosion usually occur simultaneously, so we also studied the corrosion resistance of the coating PCF in a 3.5 wt.% NaCl solution via electrochemical impedance spectroscopy (EIS) measurement. It can be seen in Fig. 8a that those samples with amorphous coatings showed remarkably larger arc diameter than that without amorphous coating, indicating a sufficiently high corrosion resistance compared with conventional antifouling Cu-based alloys used in marine industry. For example, the overall impedance value of the coating PCF is nearly 45 times higher than that of the CuAl alloy substrate (Fig. 8b). This result highlights the critical role of Fe-based amorphous coating in the design of anti-fouling and anti-corrosion coatings. Therefore, the coating PCF with dual-modifications by both Cu_2_O and FAS exhibits a good integration of outstanding corrosion resistance and anti-fouling properties, and could be a promising coating in marine applications.

## Conclusions

In this work, we fabricated a robust bioinspired anti-fouling Fe-based amorphous coating via dual surface modifications, with Cu_2_O nanofibers and superhydrophobic FAS layer. The modified amorphous coating (i.e., PCF) exhibits excellent antifouling properties, as evidenced by high antibacterial ratio (99.8%) against *Pseudomonas aeruginosa*, and good resistance to adhesion of bovine serum albumin protein (87%) and algae adhesion (98.6%), which is much better than the unmodified amorphous coating and the coatings with only a single modification (i.e., only with Cu_2_O layer or only with FAS layer ). The outstanding antifouling properties of the coating PCF originate from the synergistic effect of Cu_2_O (killing effect) and superhydrophobic surface (repelling effect). Therefore, the current work opens up an alternative avenue for designing durable anti-fouling metal-based coatings for marine applications.

## Materials and methods

### Fabrication of Fe-based amorphous coatings

The Fe_43.7_Co_7.3_Cr_14.7_Mo_12.6_C_15.5_B_4.3_Y_1.9_ (at. %) amorphous powders were prepared by gas atomization^43^, and the powders of 30–55 μm in diameter were sieved out for thermal spray. The micro-patterned Fe-based amorphous coatings were fabricated on a CuAl alloy substrate (Cu_78_Al_12_, in wt.%) by mask-assisted high-velocity oxygen fuel (HVOF) thermal spray system (UniCoatPro-WokaStar 610), as illustrated in Fig. 1b, using the optimized spraying parameters as described in our previous works^44,45^. The purpose of making a patterned structure is to establish superhydrophobic surface later on.

#### Surface modifications of the Fe-based amorphous coatings

To endow the Fe-based amorphous coating with killing effect, the Cu_2_O layer that can release Cu^+^ ions was deposited on the patterned amorphous coating by electrodeposition technique^32^. Briefly, the Cu_2_O was deposited on the coating in a mixed solution of 0.4 M CuSO_4_ and 3 M lactic acid at pH = 11 using a two-electrode system (the coating as the anode and the graphite as the cathode), following by cyclic voltammetry electro-oxidation in a 1 M KOH solution from -400 mV to 400 mV using a three-electrode configuration (Pt foil as the counter electrode and Ag/AgCl as the reference electrode). The scanning rate was 5 mV/s, and the total cycles were 4 times. After these treatments, nanofiber Cu_2_O was generated. In addition to gain superhydrophobic surface, the Cu_2_O-modified amorphous coating was immersed in a mixture solution of C_13_H_13_F_17_O_3_Si (FAS, TEXLABS technology Co., Ltd, China) and n-hexane for 2 h, followed by annealing at 85 ℃ for 20 min, upon which a superhydrophobic surface could be formed.

#### Structure characterizations

The microstructure of the coatings at various stages was characterized by X-ray diffraction (XRD, Empyrean) with Cu-Kα radiation. The surface and cross-section morphologies of the coatings were observed by a field-emission scanning electron microscope (FESEM, Sirion 200). X-ray photoelectron spectroscopy (XPS, AXIS-ULTRA DLD-600 W) measurements were performed to investigate the chemical compositions of the outmost layer, in which the sample was first etched by Ar^+^ ion for about 30 s to eliminate possible surface contaminants. The C 1 s peak at 284.8 eV was used to calibrate the XPS spectrum.

#### Wettability testing

The wettability of the coatings prepared was evaluated by water contact angle measurements on a video-based optical system (Kino SL200B), five readings were achieved for each sample. The mechanical robustness of the superhydrophobic surface was assessed by both sandpaper and sand particle abrasion tests^46,47^. For the sandpaper abrasion experiments, the coating surface was placed onto 80# mesh sandpaper with a constant load of 100 g and slowly moved at a speed of 5 cm/s for 15 cm along both the horizontal and vertical directions. For the sand abrasion tests, the Al_2_O_3_ sand particle with diameter of 0.7 to 0.85 mm continuously impinged on the coating surface from a height of 0.5 m. After the mechanical treatments, the wettability was evaluated again.

#### Anti-algal-adhesion assays

The antifouling property of the coatings with different modifications was evaluated by anti-algal-adhesion tests. Diatom *Nitzschia closterium f.minutissima* was cultivated in F/2 culture medium under a 12:12 h light–dark cycle at 20 ℃. 4 ml of *Nitzschia closterium f. minutissima* suspension was added to a 24-well microplate containing as-modified coatings, and cultured for 15 days and 30 days, respectively. The non-modified amorphous coating is the control. Then, the coatings were taken out and gently rinsed with PBS solution to remove the unattached diatom. The attached diatom was observed with a fluorescence microscope and counts were made by 5 random fields of views of each coating. Each coating was tested in triplicates.

#### Anti-protein-fouling assays

The maximum absorption wavelength of protein solution with standard concentration was determined by spectrophotometer, and the standard curve was obtained according to the absorbance of protein solution with different standard concentration at the maximum absorption wavelength. The sample was soaked in the 2 ml bovine serum albumin (BSA) protein solution (0.5 mg/ml) for 1 day, then taken out and cleaned with PBS solution. The cleaned sample was put into a 2 ml PBS solution and kept for 1 day, and then the absorbency was tested at *λ* = 280 nm. The concentration was calculated according to the standard curve, the resistance to protein adhesion is evaluated by the amount of adhesive protein divided by the initial content. Triplicate samples were tested.

#### Antibacterial assays

The antibacterial assays were conducted according to the National Standard of China GB/T 2591^48^*,* in which a marine bacteria *Pseudomonas aeruginosa* was used. The non-modified coating was used as the control group. The bacterial cultivation was carried out in 2216E liquid medium. The 2216E medium inoculated *Pseudomonas aeruginosa* was autoclaved at 121 °C for 20 min followed by air cooling at 37 °C. Bacteria were diluted with PBS solution at a concentration of 10^6^ CFU/mL. 100 μL of bacterial suspension was introduced to the as-modified coating surfaces. The bacterial biofilm was grown at 37 °C for 48 h, afterwards, the coating was taken out and washed for 3 times to remove the unattached biofilm. Planktonic bacteria were serially diluted and then placed onto the 2216E agar plates (100 μL solution) for succedent evaluation. The 2216E agar plates were incubated at 37 °C for 24 h, and then bacterial numbers were counted. Each coating was tested in three times. To calculate the Cu ion release rate, the concentration of Cu^+^ ions in the solution was measured using inductively coupled mass spectrometer (ICP-MS) after different exposure days. The side and back surfaces of the coatings were sealed by epoxy resin to expose their coating surface only (1 cm^2^).

#### EIS measurement

The corrosion resistance of the coatings was evaluated by electrochemical impedance spectroscopy (EIS) technique. The EIS tests were conducted in a 3.5% NaCl solution with an electrochemical workstation (Chenhua 760E) using a three-electrode system, wherein the counter electrode (CE) was a platinum plate, the reference electrode (RE) was a saturated Ag/AgCl electrode and the working electrode (WE) was the sample. The EIS was recorded from 10^5^ to 10^–2^ Hz at an amplitude of 10 mV under the open circuit potential. All electrochemical tests were repeated for three times.
